# Experimental Evaluation of the Thermal Polarization in Direct Contact Membrane Distillation Using Electrospun Nanofiber Membranes Doped With Molecular Probes

**DOI:** 10.3390/molecules24030638

**Published:** 2019-02-12

**Authors:** Sergio Santoro, Ivan Vidorreta, Isabel Coelhoso, Joao Carlos Lima, Giovanni Desiderio, Giuseppe Lombardo, Enrico Drioli, Reyes Mallada, Joao Crespo, Alessandra Criscuoli, Alberto Figoli

**Affiliations:** 1 Institute on Membrane Technology (ITM-CNR), via P. Bucci 17/C, 87036 Rende (CS), Italy; s.santoro@campus.fct.unl.pt (S.S.); e.drioli@itm.cnr.it (E.D.); 2 Institute of Nanoscience of Aragon (INA) and Department of Chemical, Engineering and Environmental Technology, University of Zaragoza, C/Mariano Esquillor, s/n, I+D+i Building, 50018 Zaragoza, Spain; ivan_nezumi@hotmail.com (I.V.); rmallada@unizar.es (R.M.); 3 LAQV, REQUIMTE, Departamento de Química, Faculdade de Ciências e Tecnologia, Universidade Nova de Lisboa, 2829-516 Caparica, Portugal; imrc@fct.unl.pt (I.C.); lima@fct.unl.pt (J.C.L.); jgc@fct.unl.pt (J.C.); 4 Istituto di Nanotecnologia (CNR - NANOTEC), Via P. Bucci 31c, 87036 Rende (Cs), Italy; giovanni.desiderio@cnr.it; 5 Istituto per i Processi Chimico-Fisici, CNR-IPCF, Viale F. Stagno D’Alcontres 37, 98158 Messina, Italy; giuseppe.lombardo@cnr.it

**Keywords:** membrane distillation, thermal polarization, electrospinning, molecular probes

## Abstract

Membrane distillation (MD) has recently gained considerable attention as a valid process for the production of fresh-water due to its ability to exploit low grade waste heat for operation and to ensure a nearly feed concentration-independent production of high-purity distillate. Limitations have been related to polarization phenomena negatively affecting the thermal efficiency of the process and, as a consequence, its productivity. Several theoretical models have been developed to predict the impact of the operating conditions of the process on the thermal polarization, but there is a lack of experimental validation. In this study, electrospun nanofiber membranes (ENMs) made of Poly(vinylidene fluoride) (PVDF) and doped with (1, 10-phenanthroline) ruthenium (II) Ru(phen)_3_ were tested at different operating conditions (i.e., temperature and velocity of the feed) in direct contact membrane distillation (DCMD). The temperature sensitive luminophore, Ru(phen)_3_, allowed the on-line and non-invasive mapping of the temperature at the membrane surface during the process and the experimental evaluation of the effect of the temperature and velocity of the feed on the thermal polarization.

## 1. Introduction

The lack of sufficient available fresh water resources is one of the major pervasive problems afflicting people throughout the world. In fact, more than one billion people lack access to drinking water and 3,900 children a day die from diseases transmitted through unsafe water [1].

In this scenario, membrane technology plays a key role in order to meet water needs by wastewater treatment and desalination [2,3]. Several membrane-based technologies, such as reverse osmosis (RO), nanofiltration (NF), ultrafiltration (UF), and microfiltration (MF), are currently being used to convert seawater, brackish, and wastewater into potable water [4]. Nowadays, ca. 50% of the world’s total desalination capacity is based on membranes using the concept of reverse osmosis [5]. Membrane distillation (MD) is a promising technology involving a microporous hydrophobic membrane that guarantees the transport of a considerable amount of water vapor through its void space, disallowing the permeation of water in liquid form containing dissolved non-volatile compounds (i.e., salts) [6]. MD presents several advantages, such as the use of low grade waste heat; the production of ultra-pure products; and, in particular, the possibility to treat the mixtures with concentrations beyond the operational limits of the conventional pressure-driven membrane processes (i.e., RO) [7]. The slow growth of membrane distillation has been related to the unavailability of appropriate membranes for MD applications and limited investigations carried out on module and processes design [8]. In particular, fouling, thermal polarization, and membrane wetting are considered major drawbacks in the application of MD technologies.

However, thanks to the recent and growing extensive research activities carried out in various areas of MD, the process has become much more attractive due to the availability of membranes with an enhanced performance and the possibility to utilize alternative energy sources [8].

In the last years, much effort has been devoted to developing membranes with tailored properties designed for MD applications. Poly(vinylidene fluoride) (PVDF) has been extensively studied as a potential candidate in MD with regard to its outstanding properties, such as its mechanical, thermal, and chemical resistance, and hydrophobicity. PVDF is also advantageous because of its membrane forming property and solubility in a wide range of solvents, which are severe limitations for other common hydrophobic polymers, such polytetrafluoroethylene (PTFE) and polypropylene (PP), to be used in traditional phase inversion techniques [9].

In the past few years, nanotechnology has been gaining momentum in membrane preparation, fabrication, and modification: nanoparticles and nanofibers are widely employed to enhance the basic properties of membrane materials or to add novel properties and functionalities to the membrane [10]. For instance, electrospun polymeric nanofibers exhibit appealing properties appropriate for MD applications, such as a high surface roughness combined with an excellent porosity and mechanical robustness [11]. In fact, electrospun nanofiber membranes (ENMs) consist of high flux and thermally efficient membranes due to their high porosity, above 80%, with interconnected structures and a high hydrophobicity [12,13].

In recent studies, luminophores, molecules emitting light as a consequence of the exposure to an excitation source, have been proposed as an innovative technology for monitoring membrane processes. In particular, tris (1, 10-phenanthroline) ruthenium (II) (Ru(phen)_3_) was immobilized in different membranes, allowing in-situ and non-invasive monitoring and/or mapping of oxygen and temperature on the membrane surface [14,15,16]. Moreover, transition metal polypyridyl complexes such as Ru(phen)_3_ are successfully employed in the development of fibre-optic oxygen sensors for oceanography due to their non-sensitivity towards the salinity [17]. Moreover, PVDF electrospun nanofibers doped with Ru(phen)_3_ were successfully applied by the authors in direct contact membrane distillation (DCMD), and obtained a membrane that, by using a specifically designed membrane module, allowed them to map the temperature on the membrane’s surface on-line [18]. In particular, as proof of concept, a map of the temperature distribution on the membrane surfaces at both feed and permeate sides was obtained when working at fixed operating conditions.

On the basis of the positive results obtained, the aim of the present study was to continue the investigation, focusing on the optimization and evaluation of the effects of the operating conditions on the performance of the MD process. PVDF membranes prepared via electrospinning loaded with the Ru(phen)_3_ probe were tested in DCMD by varying the temperature and the velocity of the feed. The luminophore allowed us to non-invasively map the temperature on the membrane surfaces, whereas an IR-camera monitored the bulk temperature of the water streams, experimentally demonstrating the evident effect of both investigated parameters (temperature and feed velocity) on the driving-force and, as a consequence, on the performance of the process.

## 2. Materials and Methods

### 2.1. Materials

Poly (vinylidenefluoride) Solef^®^ 6012(PVDF) was kindly supplied by Solvay Specialty Polymers (Bollate, Italy). Solvents such as N,N Dimethylformamide (DMF, 99.8%, Sigma Aldrich, Spain) and acetone (Panreac, Spain) were employed to dissolve PVDF. Lithium chloride (LiCl, Fisher Chemicals, Spain) was employed as an additive. The luminophore tris(phenantroline)ruthenium(II) chloride (Ru(phen)_3_) was purchased from Sigma–Aldrich.

### 2.2. Membrane Preparation by Electrospinning

The method for the preparation of PVDF electrospun membranes was widely described in a previous paper [18]. Briefly, the molecular probe Ru(phen)_3_ (0.83 wt% with respect to the PVDF) and the additive LiCl (0.43 wt% with respect to PVDF) were firstly dispersed in the mixture of DMF/Acetone. It has been demonstrated that LiCl plays a key role in adjusting the viscosity and, in particular, the conductivity of the solution, allowing for the preparation of defect-free fibers [18,19,20,21]. In fact, LiCl plays a key role in order to favor the formation of nano-fibers by generating a higher charge density on the surface of the charged jet, thus facilitating the electrospinning of the polymeric solution [19].

Subsequently, the PVDF dope solution was prepared by solubilizing the polymer at a concentration of 10 wt% in the blend of DMF/Acetone (6:4 wt:wt) containing LiCl and Ru(phen)_3_ by stirring overnight at a temperature of 70 °C.

**Figure 1:**
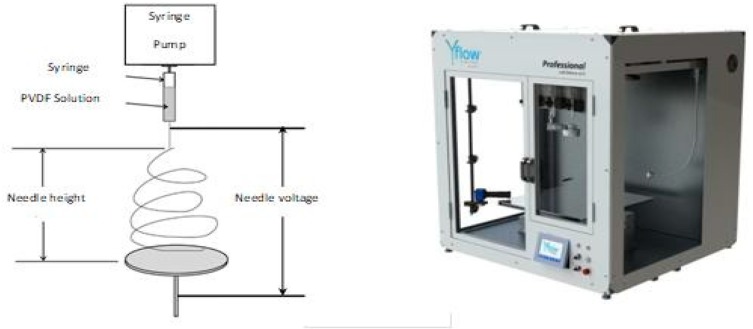
Scheme and picture (http://www.yflow.com) of the electrospinning set-up.

After cooling at room temperature, the PVDF solution was transferred in a syringe and electrospun by means of a Yflow 2.2 D500 electrospinner with a 20-gauge needle used to obtain the fibers (Figure 1). Table 1 summarizes the optimized operating conditions of the electrospinning process. These operating conditions were selected in order to favor the evaporation of the solvents, to avoid the formation of beads, and to obtain a self-consistent membrane.

Finally, the produced membranes were submitted to thermal post-treatment (overnight at 130 °C) in order to facilitate the linkage of the network made of PVDF nanofibers.

### 2.3. Membranes Characterization

The morphology of the ENM PVDF membrane was observed using a Scanning Electron Microscope (SEM) (Zeiss-EVO MA10 instrument, Milan, Italy). The membrane was coated with a thin gold layer by sputtering using a Quorum Q150 RS sputter (Quorum Technologies, Laughton, East Sussex, UK) in order to enhance the membrane conductivity and prevent electrical charging.

The structure of the ENM PVDF was also observed by using a Leica SP8 confocal laser scanning microscope (Leica Microsystems GmbH, Wetzla, Germany). The microscope was equipped with an Argon laser (65 mW) for the excitation of the Ru(phen)_3_ immobilized in PVDF nano-fibers at 458 nm. The Leica SP8-Spectral Scan-Head allows the acquisition of 2D images in the focal plane by scanning the laser beam focused by a Leica HCX IRAPO 25X/095 NA IRAPO water immersion objective with a working distance of 2.5 mm. Pictures were collected with a frequency of 400 Hz and a pixel to voxel size ratio of 100 nm with a frame acquisition time ranging from 1.5 s up to 100 s (from 500 × 500 pixels to 4096 × 4096 pixels, respectively). The emission rising from the membrane was collected in the epi-detection by a photomultiplier tube (PMT) set in the range of 520–750 nm by the use of the filter-free spectral detecting system.

The hydrophobicity of PVDF ENM was evaluated by measuring the contact angle with distilled water using a CAM 200 contact angle meter (KSV Instruments, Finland), according to the sessile drop method at ambient temperature.

Membrane porosity was evaluated by the gravimetric method consisting of weighing the membrane in dry and wet conditions after immersion for 24 h in kerosene [22]. The porosity was estimated according to the following equation:

(1) $$P=\frac{\frac{w_{h}-w_{d}}{\rho_{w}}}{\frac{w_{d}}{\rho_{PVDF}}+\frac{w_{h}-w_{d}}{\rho_{w}}};$$

where w_h_ is the weight of the wet membrane; w_d_ is the weight of the dry membrane; ρ_w_ is the kerosene density (0.82 g cm^−3^); and ρ_PVDF_ is the PVDF density (1.72 g cm^−3^) [22].

The pore size was determined using a PMI Capillary Flow porometer (Porous Materials Inc., Itacha, NY, USA), according to the procedure reported in the literature [23]. Briefly the PVDF ENM was immersed for 24 h in Porewick (16 dyne cm^−1^) and then tested in the membrane module of the porometer using a wet-up/dry-up method programmed by the software Capwin.

The liquid entry pressures (LEPs) of the PVDF ENM was evaluated by accommodating a membrane sample in a chamber filled-up with 200 mL of DI water at 20 °C: the pressure of N_2_ in the chamber was increased at a constant rate of 0.1 bar every 10 min until the water permeated through the membrane. 

### 2.4. DCMD Experiments and on-Line Monitoring of the Membrane Surfaces Temperature

**Figure 2:**
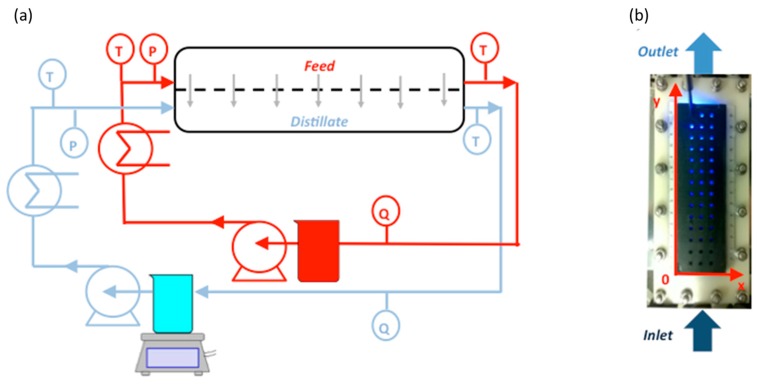
(**a**) Scheme of the set-up employed for DCMD test equipped with sensors for monitoring the temperature (T), the flow rate (Q), and the pressure (P) of the feed and the distillate; (**b**) Picture of the membrane module developed for optical observation.

DCMD experiments were performed using the set-up shown in Figure 2a. The feed and permeate temperatures were controlled by a heater and a cooler, respectively. The temperatures of both feed and distillate streams were monitored by means of Platinum thermistors (PT100, Delta OHM, accuracy ± 0.1 °C) placed in proximity to the inlets and outlets of the membrane module. The distillate stream was kept at an inlet temperature of ca. 18–19 °C, whereas the feed was heated-up to 40, 50, and 60 °C.

The streams were fed co-currently by means of two peristaltic pumps (Masterflex^®^ 7518-10). No mesh/spacer was used at both feed and permeate sides. At the feed side, the channel for the stream flow was thicker (1.7 cm) than the distillate side. Larger channel gaps were needed to investigate the effect of the feed velocity (Reynolds) without increasing the pressure drops. 

The feed (distilled water) flow rate varied from 25 L·h^−1^ to 75 L·h^−1^, corresponding to feed velocities ranging from 0.008 m·s^−1^ to 0.024 m·s^−1^, whereas the distillate flow rate was fixed at 25 L·h^−1^ and the corresponding velocity was 0.055 m·s^−1^. At 60 °C, the Reynolds value at the feed side (equivalent diameter of 2.54 cm) ranged from 417 to 1250, while at the distillate side (equivalent diameter of 0.317 cm), it was 178 at 20 °C. The trans-membrane flux was determined by weighing the distillate produced using an analytical balance (Europe 6000, Gibertini, Italy).

The PVDF electrospun membrane containing molecular probes was placed in a membrane module made of Nylon (overall size: 16 cm × 5.5 cm; effective size: 15 cm × 4 cm; active area: 60 cm^2^) equipped with a polymeric transparent window in the near-UV/visible region, allowing the optical observation (Figure 2b). In fact, it was possible to record the emission rising from the molecular probe immobilized in the PVDF membrane by means of a bifurcated fiber-optical bundle transparent in the UV/Visible range (Ocean Optics). One branch of the bifurcated fiber-optical was connected to LED (emitting at 450 nm) for the excitation of Ru(phen)_3_ and the other branch to a spectrofluorimeter (Avantes) to collect the emission from the molecular probe. The membrane module was equipped with a special non reflective black cover with 42 holes, allowing us to fix the spot of spectroscopic acquisition on the membrane surface, allocating the optical fiber at 90° with respect to the membrane surface. The 42 spots of observation are displaced in three rows and 14 columns spaced 1 cm apart from each other. The acquisition time of spectra was set at 0.2 s and 10 spectra were averaged for each measurement in order to obtain the maximum signal to noise ratio.

The calibration curves were obtained for each of the 42 spots by plotting the amplitude of the phosphorescence emitted by the molecular probe at 572 nm as a function of the membrane temperature. In this phase, the two water streams (feed and distillate) were kept at the same temperature, guaranteeing a homogeneous temperature in the membrane module and avoiding heat-loss through the membrane. The surface adjacent to the feed was calibrated at a temperature ranging from 40 °C to 60 °C, whereas the side of the membrane exposed to the distillate side was calibrated at 18 °C, 25 °C, and 35 °C.

As expected, in all the cases, the intensity of the emission of Ru(phen)_3_ linearly decreased by increasing the temperature: i.e., the phosphorescent emission was higher in proximity of the outlet rather than in the inlet membrane module. In fact, the Arrhenius-type dependence of the intensity of the emission with respect to the temperature attributed to the thermal-driven non-radiative decay of the excited state is usually well-approximated to a linear trend at low temperature [24].

The bulk temperature of water streams (feed and distillate) was monitored using an IR CAMERA (model FLIR E40, sensitivity of 0.07 °C at 30 °C, resolution of 160×120 pixels, spectral range from 7.5–13 µm).

## 3. Results

### 3.1. Membrane Characterization

**Figure 3:**
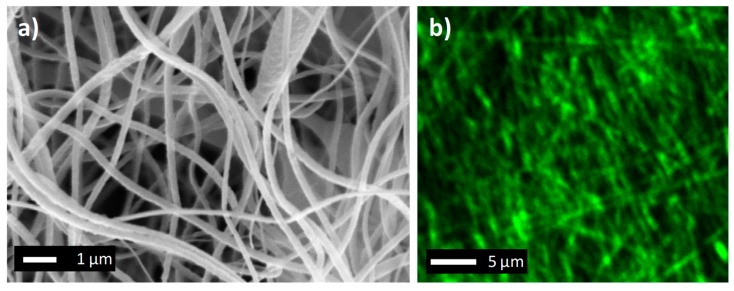
PVDF membrane: (**a**) SEM picture; (**b**) Confocal microscope picture.

The PVDF membrane produced consisted of a 3D network of fibers, as shown in Figure 3a. PVDF electrospun nanofibers presented a diameter of around 232 ± 48 nm, whereas the membrane thickness was 40 μm. The network of nanofibers produced a microporous structure with desired properties in MD application. In fact, the membrane presented a porosity of 89 ± 1% and a mean pore size of 0.75 ± 0.04 μm. These properties allow the PVDF ENM to minimize its resistance to vapor transport, whereas its high hydrophobic character (contact angle of 115 ± 4°) avoids the permeation of liquid water, preventing the wetting of pores till a hydrostatic pressure of 1 bar (LEP).

The 3D network of the fibers was also visible by observing the PVDF ENM with the Scanning Electron Microscope (Figure 3a) and confocal microscope (Figure 3b). In fact, fibers exposed to light excitation emitted a considerable amount of light due to the photochemical activity of Ru(phen)_3_ widely employed as a molecular probe because of its outstanding properties, such as its brightness and relatively long lifetime. The picture evidences the presence of the nanofibers and sub-micrometric channels for water vapor transport.

**Figure 4:**
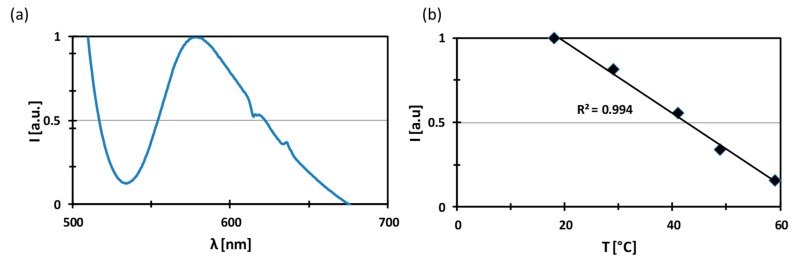
(**a**)Emission of PVDF ENM excited at 450 nm at 18 °C; (**b**) Effect of the temperature on the intensity of the emission at 572 nm.

The phosphorescent spectra collected in Figure 4a confirmed the emission of the PVDF ENM due to the immobilization of the complex of Ru in the nanofibers. In fact, the membrane produces an intense and broad emission in the visible region, with a maximum of emission at 572 nm. The intensity of the emission of the PVDF ENM at 20 °C was stable over the testing campaign (experimental error below 3% for two months), evidencing the chemical stability of the molecular probe encapsulated in the polymeric nanofibers and the absence of phenomena of releasing of the molecular probes from the polymeric matrix to the water streams. This was confirmed by the absence of [Ru(phen)_3_]^2+^ in both feed and distillate streams since no luminescent activity was observed by analyzing the water streams. This was attributed to strong electrostatic attraction forces between the positively charged ([Ru(phen)_3_]^2+^) complex and the negatively charged fluoropolymer (PVDF).

Moreover, the intensity of the emission dramatically decreased when increasing the temperature, as expected. Figure 4b shows the linear dependence of the intensity of the emission with respect to the temperature. This effect is due to the fact that the increase of the temperature favors internal conversion of the energy to vibrational energy, causing a decrease of the photochemical activity [25].

### 3.2. DCMD Performance

**Figure 5:**
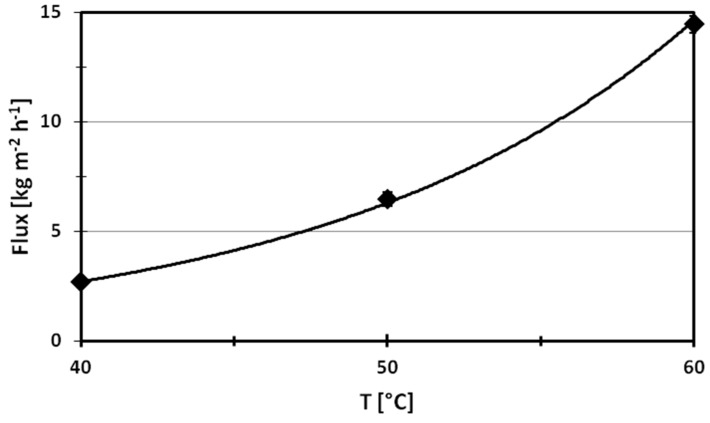
Effect of the feed temperature on the flux of PVDF ENM in DCMD process at a feed velocity of 0.008 m·s^−1^.

Figure 5 summarizes the effect of the feed temperature on the performance of the PVDF electrospun nanofibers membrane in terms of flux. The trans-membrane flux is significantly affected by the feed temperature, following an Arrhenius trend. In particular, the flux drops from 14.4 kg·m^−2^·h^−1^ to 2.7 kg·m^−2^·h^−1^ by reducing the temperature from 60 °C to 40 °C. This is mostly attributed to the fact that the increase of the temperature of the feed produces a positive effect on the driving-force of DCMD processes. In fact, according to the Antoine equation, the vapor pressure increases exponentially with temperature, therefore exponentially affecting the productivity of the process [26]. 

In all tests, the flux varied during the first hour of operation, and was then stable over a running time of 3 h. In fact, the experimental error of the flux was evaluated to be lower than 3% by measuring the flux every 15 min, and then confirming the stability of PVDF ENM.

**Figure 6:**
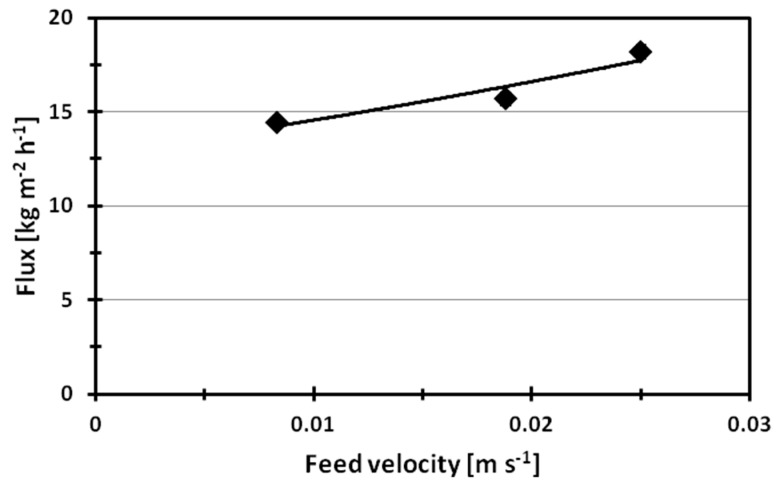
Effect of the feed velocity on the flux of PVDF ENM in DCMD process at a feed temperature of 60 °C.

A positive effect of the flux was also observed by increasing the velocity of the feed. In fact, the flux increased from 14.4 kg·m^−2^·h^−1^ to 18.2 kg·m^−2^·h^−1^ by speeding up the feed stream from 0.008 m·s^−1^ to 0.024 m·s^−1^ (Figure 6).

**Figure 7:**
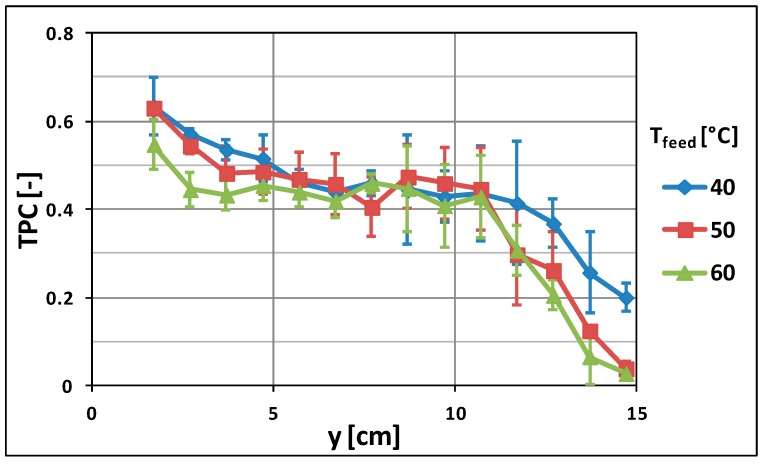
TPC along the membrane module: effect of feed temperature (v_feed_ = 0.008 m·s^−1^).

**Figure 8:**
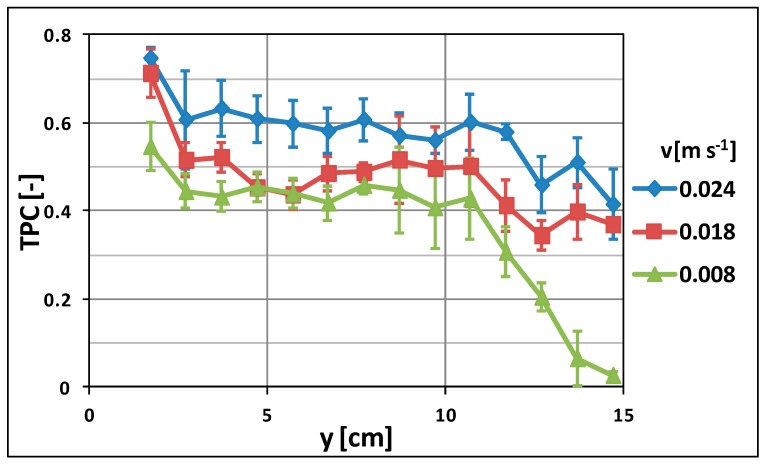
TPC along the membrane module-effect of feed velocity (T_feed_ = 60 °C).

This is due to the fact that the increase of the feed velocity enhanced mixing in the flow channel and reduced the boundary layer from the feed stream to the membrane surface as a consequence, improving the heat transfer coefficient [27,28]. In fact, the increase of the feed velocity of a factor of 3 increased the flux ca. 27%. It has to be pointed out that the indicated feed velocity values, in Figure 5, Figure 6, Figure 7 and Figure 8, were calculated by considering the inner cross section of the module (17 × 50 mm), as fully developed profiles. The feed velocities investigated correspond to Reynolds values ranging from 417 to 1250. As a rough analysis, the trend of the trans-membrane flux follows that of Re^0.405^ quite well when the Reynolds value is ≥1000, which indicates a laminar regime.

Even though it is difficult to compare the membrane performance with literature data, due to the fact that the operating conditions and the module designs reported in the literature are quite different to each other, it is possible to notice in Table 2 that the developed PVDF membrane prepared via electrospinning presented comparable permeability values with respect to commercial unsupported membranes, as taken from [29]. In particular, in the table, the most commonly used hydrophobic materials are reported. 

### 3.3. Evaluation of the Effect of the Feed Temperature and Velocity on Thermal Polarization

Membrane distillation is a thermally-driven separation process, in which the driving force is the vapor pressure difference created across the membrane. In particular, in DCMD processes, heat is transported through the membrane by two different mechanisms: latent heat of evaporation and conductive heat transported across the membrane, which negatively affects the driving force, representing heat loss. The evaporation process is also a cause of temperature polarization, leading to a temperature reduction at the feed-membrane interface due to feed evaporation and to a temperature increase at the membrane-permeate side as a consequence of the vapor condensation. Furthermore, to reach the membrane surface, heat moves from the bulk of the feed through the boundary layer, and as a consequence, the temperature at the membrane surface is lower than that in the bulk phase. The same happens at the cold side, where the vapor transported from the feed condenses: due to the presence of the boundary layer, the temperature at the membrane surface is higher than that of the bulk cold stream. To take into account both aspects, the thermal efficiency of DCMD processes is quantified by the Temperature Polarization Coefficient (TPC) evaluated as the ratio between the actual driving force (across the membrane) and the theoretical driving force (across the streams bulk) and is expressed mathematically as follows:

(2) $$\mathrm{TPC}=\frac{T_{F,M}-T_{D,M}}{T_{F}-T_{D}}$$

where T_D_ is the temperature of the bulk distillate; T_D,M_ is the membrane temperature at the distillate side; T_F_ is the temperature of the bulk feed; and T_F,M_ is the membrane temperature at the feed side [29,30]. The higher the TPC, the better the process performance. TPC can vary between 1 (when no polarization occurs) and 0 (in the case of complete polarization). Usually, the value of TPC lies between 0.4–0.7 for DCMD [31].

The temperature on the membrane surfaces, i.e., T_F,M_ and T_D,M_, was evaluated by processing the phosphorescent emissions of Ru(phen)_3_ through the holes displaced in three rows (*x*-axis) and 14 columns (*y*-axis) spaced 1 cm apart (Figure 2b); whereas the temperatures of the bulk streams, i.e., T_F_ and T_D_, were monitored by the IR camera with a spatial resolution of 0.1 cm. The 10 values of the temperatures of the feed or distillate corresponding to the area of observation of the phosphorescent measurements were averaged, and in this case, a map with a resolution of 1 cm was also obtained. Since the measurements of T_F_ and T_D_ have been acquired with a spatial resolution 10 times greater than the temperature on the membrane surfaces, we extracted an average value of T_F_ and T_D_ for every 1 cm.

Finally, the thermal efficiency of the DCMD process was evaluated on each of the 42 spots and the behaviour of TPC along the membrane module (*y*-axis) was then extracted by averaging its values along the *x*-axis.

Figure 7 shows the trend of the mean value of TPCs along the membrane module at different inlet feed temperatures (T_feed_ = 40, 50, and 60 °C).

Figure 7 evidences that the thermal efficiency of the process dramatically decreases along the membrane. In fact, in all the cases, the TPC in proximity to the inlet of the membrane module (low value of y) is much higher than the one observed close to the membrane outlet (high value of y).

As already pointed out, this effect is due to heat spent to produce vapor and transported by the membrane as a consequence of the DCMD process, as well as some heat loss towards the environment that reduces the feed temperature. In addition, Figure 7 shows the key role of the feed temperature in the thermal efficiency of the process depicting the tendency of the TPC. This is due to the fact that high temperatures of the feed imply an increasing of both convective and conductive heat fluxes through the membrane, causing a more pronounced temperature gradient between the bulk of the streams and the correspondent membrane surfaces [32].

Figure 8 summarizes the effect of the feed velocity on the thermal efficiency of the DCMD process by showing the trend of TPC at different feed velocities. The impact of the feed velocity on the thermal efficiency of the process is evident: the TPC increased by increasing the feed velocity. This is a result of the mixing effect and reduction of the boundary layer resistance obtained when working at higher Reynolds values, which moved from 417 (at 0.008 m/s) to 1250 (at 0.024 m/s) [33].

At low values of the feed velocity, the transfer of heat from the bulk to the membrane surface is hindered by the boundary layer and low values of the TPC are observed as a consequence. On the other hand, an increase in feed velocity improves the Reynolds number of the streams, favors the mixing of the fluid, and decreases the thickness of the boundary layer [34]. The final effect is the reduction of the difference between the temperature at the membrane surface and the bulk and, as a consequence, an increasing of the TPC. It has to be noticed that TPC decay at the extremities of the membrane module was observed, especially at low velocities. This trend could be attributed to entrance and exit effects: the fluid enters the module from a central hole (i.d., 8 mm) and is then spread on the membrane surface for the first centimeters, reducing its velocity. Following this, it moves at a constant velocity (fully developed profile), until it reaches the last part of the module, where it has to converge towards the exit central hole, further reducing its velocity and, then, the TPC.

## 4. Conclusions

PVDF ENM doped with Ru(phen)_3_ was tested in DCMD processes by varying the temperature and the velocity of the feed stream. The experiments show the crucial role of the temperature of the feed, which affects the driving-force of the process. In fact, the flux increases from 2.7 kg·m^−2^·h^−1^ to 14.4 kg·m^−2^·h^−1^ by raising the temperature of the feed from 40 °C to 60 °C. A positive effect on the flux was also observed by increasing the velocity of the feed from 0.008 m·s^−1^ to 0.024 m·s^−1^, attributed to the reduction of the thermal polarization phenomena achievable by increasing the Reynolds number of the feed from 417 to 1250.

The immobilization of Ru(phen)_3_ as temperature-sensitive luminophore allowed us to study the dissipation of heat along the membrane module, producing a scattering of the temperature on the membrane surfaces. The highest flux was obtained at a feed temperature of 60 °C and velocity of 0.024 m·s^−1^. In these conditions, the TPC varied between 0.75 and 0.4 at the inlet and outlet of the module, respectively. In particular, it was observed that the TPC was basically constant along the membrane module (TCP~0.6 between 2 cm and 12 cm), whereas it dropped in proximity of the inlet and the outlet of the membrane module, due to entrance/exit effects.

This work confirmed that the proposed approach allows us to calculate experimentally, in a non-invasive way, the thermal polarization of the system, as a function of the main operating parameters of the DCMD process. In fact, the employment of optical techniques based on the use of the molecular probe for monitoring the temperature at membrane surfaces and of an IR-camera for the detection of the temperature of the bulk streams can be considered a powerful tool to design high-performance membrane modules for DCMD and to optimize the operating conditions of the process.

## Figures and Tables

**Table 1:** Operating conditions of the electrospinning process.

Flow rate (mL h^−1^)	1
Needle height (cm)	15
Needle voltage (kV)	+16
Collector voltage (kV)	−2

**Table 2:** Comparison of the permeability values among the membrane produced in this work and those reported in ref. [29].

Producer-Trade Name	Material	Feed	LEP (bar)	d_p_ (µm)	Ԑ (%)	Permeability (kgh^−1^m^−2^ bar^−1^)	Operating Conditions
This work	PVDF	Distilled water	1.0	0.75	89	237	T_f_ = 60 °C, T_d_ = 20 °C, v = 0.024 m·s^−1^
Millipore Durapore HVHP	PVDF	Seawater	2.0	0.45	75	214	T_f_ = 60 °C, T_d_ = 45 °C v = 0.13 m·s^−1^
Membrana Accurel PP	PP	Seawater	2.5	0.2	83	237	T_f_ = 60 °C, T_d_ = 45 °C v = 0.13 m·s^−1^
Donaldson Tetratex	PTFE	Seawater	9.9	0.2	83	259	T_f_ = 60 °C, T_d_ = 45 °C v = 0.13 m·s^−1^
